# Application of Pd-Based Membrane Reactors: An Industrial Perspective

**DOI:** 10.3390/membranes8040101

**Published:** 2018-11-01

**Authors:** Emma Palo, Annarita Salladini, Barbara Morico, Vincenzo Palma, Antonio Ricca, Gaetano Iaquaniello

**Affiliations:** 1 KT—Kinetics Technology S.p.A., Viale Castello della Magliana 27, 00148 Rome, Italy; e.palo@kt-met.it; 2 Processi Innovativi srl, Via di Vannina 88, 00156 Rome, Italy; salladini.a@processiinnovativi.it (A.S.); morico.b@processiinnovativi.it (B.M.); 3 Department of Industrial Engineering, University of Salerno, via Giovanni Paolo II 132, 84084 Fisciano, SA, Italy; vpalma@unisa.it (V.P.); aricca@unisa.it (A.R.)

**Keywords:** Pd-based membrane, hydrogen, closed architecture, open architecture, gas to liquid, propylene

## Abstract

The development of a chemical industry characterized by resource efficiency, in particular with reference to energy use, is becoming a major issue and driver for the achievement of a sustainable chemical production. From an industrial point of view, several application areas, where energy saving and CO_2_ emissions still represent a major concern, can take benefit from the application of membrane reactors. On this basis, different markets for membrane reactors are analyzed in this paper, and their technical feasibility is verified by proper experimentation at pilot level relevant to the following processes: (i) pure hydrogen production; (ii) synthetic fuels production; (iii) chemicals production. The main outcomes of operations in the selected research lines are reported and discussed, together with the key obstacles to overcome.

## 1. Introduction

Membrane reactors are currently increasingly recognized as an effective way to replace conventional separation, process, and conversion technologies for a wide range of applications. In particular, by taking benefit from advanced membrane materials development, they are able to provide enhanced efficiency, are very adaptable, and may have great economic potential.

On the basis of their flexibility, membrane reactors can be employed in a wide range of applications where energy saving and the enhancement of performance in terms of reactants conversion and products selectivity can lead to improved economics.

Among the different membrane reactors reported in the scientific literature [1,2,3], the palladium-based ones are the most commonly used when hydrogen is the product to be separated [4,5,6,7,8,9].

Steam reforming is the most widely application for that, since this process is strongly energy-intensive; however, the whole hydrocarbon processing industry, in principle, could take benefit from this technology, since, while enabling a substantial energy saving, it can be helpful also for the production of a highly concentrated CO_2_ stream, ready for valorization [8]. 

Accordingly, several application areas of Pd-based membrane reactors have been considered by KT—Kinetics Technology (KT): (i) pure hydrogen production; (ii) gas-to-liquid (GTL) processes; (iii) propylene production.

Natural gas (NG) steam reforming is the most widely used hydrogen production process. Currently, around 50% of the worldwide hydrogen yearly production results from this technology.

The main reaction occurring in NG steam reforming is endothermic (CH_4_ + H_2_O = CO + 3H_2_) and limited by chemical equilibrium, thereby significant hydrogen yields are achieved only with operation at high temperatures (850–900 °C). As a consequence, a portion of methane feedstock must be burned in furnaces in order to sustain the reaction heat duty. This can be responsible for a reduction of the overall process efficiency, an increase of greenhouse gas (GHG) emissions, and a dependence of the hydrogen production cost on the natural gas cost. Accordingly, the coupling of the reaction unit with hydrogen-selective membranes can represent a promising way to enhance hydrogen yield at lower temperatures, since the continuous selective removal of hydrogen from the reaction environment allows to maintain the gas mixture composition far from equilibrium, so that equilibrium conditions are not achieved, and the endothermic reactions can be carried out at a lower temperature. 

In addition, the lower thermal duty could also allow the operation of the membrane reformer with a cleaner energy source or with waste heat available from another process, instead of the high-temperature flue gases used in the furnace. Moreover, the lower operation temperature makes possible to use cheaper steel alloys for the fabrication of the reforming tube instead of the expensive materials currently used to withstand the high operating temperatures of conventional steam reforming plants.

With reference to the GTL processes, the main challenge of monetizing gas resources is logistical. Natural gas reserves close to markets are usually transported via pipelines. Where this is not feasible, the gas can be transported in alternative forms, such as compressed natural gas (CNG), liquefied natural gas (LNG), and GTL products, which all address this challenge by densifying the gas and reducing the transportation costs. Natural gas-to-liquid technologies have been a matter of study for many years but are considered economically not convenient, owing to the high costs of natural gas. Nevertheless, the current prospects in shale gas production as well as an increase in oil price have determined a significant difference between oil and gas prices, thus improving the economic benefits from GTL processes application and making this technology the most promising alternative for the valorization of natural gas assets, in particular in North America [10,11]. The GTL process has three main steps: (i) feedstock preparation and syngas production; (ii) Fisher–Tropsch (FT) synthesis; (iii) product upgrading. Syngas production typically involves steam reforming or an autothermal reforming reaction with pure oxygen from an Air Separation Unit (ASU); product upgrading typically involves hydrocracking processes of syncrude. The core of the technology is represented by Fischer Tropsch (FT) synthesis which requires an H_2_/CO ratio of about 2.0.

The syngas production step is the most expensive of the three processes, accounting for up to 50% of the Capital Expenditure (CAPEX). However, feed consumption is responsible for up to more than 80% of all operating costs and more than 60% of the cost of production. Therefore, there is a significant incentive for developing new technologies to decrease the capital and operating cost of syngas production units. Furthermore, since developing and constructing a large-scale GTL plant is very capital-intensive and takes years, with significant market timing and hence economic risks involved, the possibility to think of a modular design of smaller scale GTL plants is opening up opportunities to reduce risks and, at the same time, for the use of natural gas in both offshore and remote on-shore locations [12]. The development of innovative process schemes for the production of synthesis gas at lower temperatures than the traditional ones, without affecting natural gas conversion and, at the same time, with saving in terms of feed consumption and plant complexity, is crucial to assess the potentiality of distributed GTL plants. In particular, the use of membrane reactors coupled with novel routes for syngas production such as Catalytic Partial Oxidation (CPO) can be considered the basis for the development of a novel process scheme suitable for GTL applications.

With reference to the last area of the studied applications, propylene is one of the most important derivatives in the petrochemical industry, after ethylene. Worldwide, propylene is mostly produced as a co-product in steam crackers (>55%) or as by-product in Fluid Catalytic Cracking (FCC) units (around 35%). However, the high reaction temperatures of such processes, as well as the high instability of hydrocarbons, lead to formation of coke and the occurrence of side reactions that significantly impact propylene yield [13]. In order to meet the increase in the market demand, being estimated as 5% per year until 2018, several “on-purpose” technologies have been proposed as propylene sources, such as propane dehydrogenation (PDH), methanol-to-propylene (MTP) conversion, and olefin metathesis. It is widely recognized that, in the longer term, “on-purpose” propylene production technologies should be able to stabilize the supply–demand balance. 

PDH (C_3_H_8_ = C_3_H_6_ + H_2_) is a highly endothermic reaction, accordingly favored at high temperatures of operation. In these conditions, other side reactions usually can be observed, responsible for the production of lighter hydrocarbons or coke deposited on the catalyst, thereby forcing to carry out a periodic catalyst regeneration in order to recover its activity after deactivation. The two targets of decreased coke formation and increased propylene yield could be in principle achieved by integrating a low-temperature dehydrogenation reaction step with a H_2_-selective membrane [14,15] able to remove the produced hydrogen from the system and thus promoting a chemical equilibrium towards the production of propylene [16,17]. Ideally, a membrane reactor would enable the operation at a lower temperature, avoiding: (i) the search for a trade-off between conversion and selectivity; (ii) too many frequent regeneration cycles. Pd-based membranes appear as the most promising solution for the integration with the PDH reaction, owing to their significant selectivity/flux ratio and to the conventional operating temperature (400–500 °C) that is aligned with the PDH reaction operating conditions [18].

In this paper, it is reported the experience gained by KT in the design and testing of pilot facilities where membranes for hydrogen separation play a key role for the overall process performance. The results reported in the paper, being far from presenting a detailed characterization of the adopted membrane in terms of flux and permeance, aim to give a sketch of the influence of membrane integration on the overall process performance, evidencing that the experimentation at pilot level is necessary to fully understand that the operation of an industrial catalytic membrane reactor is closely linked not only to the selection of active and selective membranes and catalysts but also to the individuation of reliable procedures for a correct operation and maintenance. 

## 2. Experimental: Pilot Plants Description 

A selective membrane can be integrated with the reaction environment in two different configurations, with different benefits and drawbacks: (i) the selective membrane is in direct contact with the reaction environment/catalyst, and the reaction product is continuously removed simultaneously to its production (Integrated Membrane Reactor (IMR) or closed architecture); (ii) the selective membrane is not in direct contact with the reaction environment/catalyst but installed outside the reactor and followed downstream by another reaction unit, where the overcoming of chemical equilibrium is observed (Staged Membrane Reactor (SMR), or Reactor and Membrane Module (RMM), or open architecture) [19,20].

The level of integration of catalyst and membrane is actually important to determine the overall process yield, with the main benefit of an open architecture lying in the possibility to keep the membrane and reaction environment separate. In this way, the operating temperatures in the reactor and in the separator can be managed and optimized separately. Accordingly, for each of the studied applications, the selection between open and closed architecture was carried out on the basis of the reaction characteristics, in particular the eventual occurrence of more stressful conditions that can be detrimental for the membrane lifetime.

### 2.1. Pure Hydrogen Production

For the pure hydrogen production case, both architectures were taken into account.

**Figure 1:**
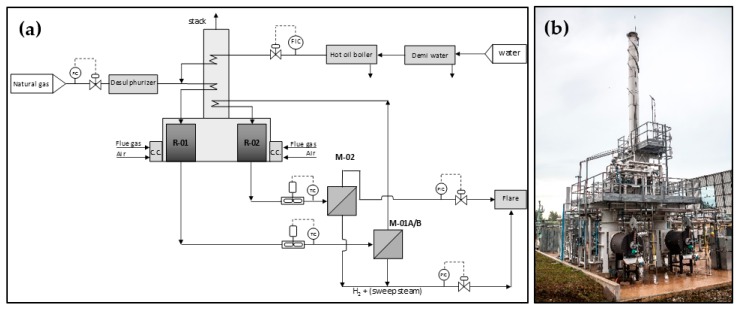
Pure hydrogen production membrane reactor in open architecture (**a**) Process scheme; (**b**) Bird-eye view.

The open architecture was tested at pilot level and at a capacity of 20 Nm^3^/h of pure hydrogen (facility available at Chieti Scalo, Italy, Italian FISR project). The process scheme of the pilot unit is reported in Figure 1 [21,22,23,24].

The plant is characterized by two-stages reformers and two membrane modules operated in the temperature range 550–650 °C and 400–450 °C, respectively. Natural gas, available at battery limits at 12 barg, was subjected to desulphurization and then mixed with the process steam. The process steam was produced separately by a real hot oil boiler, superheated in the plant convection section and routed to the first reforming reactor. Each reforming module is characterized by two main sections: (i) a radiant tube, loaded with the catalyst, and (ii) a convection section, where the recovery of heat from the flue gases, available at a temperature of 650–700 °C, was carried out for: (i) feed preheating, (ii) steam superheating.

The syngas produced in the first reformer R-01 was cooled down to the temperature suitable for membrane operation and fed to the first separation module M01A/B (0.4 and 0.6 m^2^ respectively). The retentate was recycled to the second reformer R-02, at the outlet of which a mixture with an increased amount of H_2_ in consequence of the higher feed conversion was produced. The syngas from the second reformer stage was cooled down from 650 °C to the temperature suitable for membrane operation and routed to the second separation module (M-02, 0.13 m^2^). H_2_ recovered from both membrane modules was collected and routed to the final cooling and condensate separation. The retentate from R-02 reformer was sent to the flare.

Both R-01 and R-02 were loaded with a structured foam-shaped catalyst, based on noble metals; the main reactors and catalyst characteristics are reported in Table 1.

Three different membrane modules, Pd- and Pd/Ag -based, able to operate at high temperatures (480 °C for M-01/A and M-02, 500 °C for M-01/B), were installed in the pilot unit. Their main features are reported in Table 2.

Table 3 reports the main operating conditions adopted during the experimental test on open architecture.

**Figure 2:**
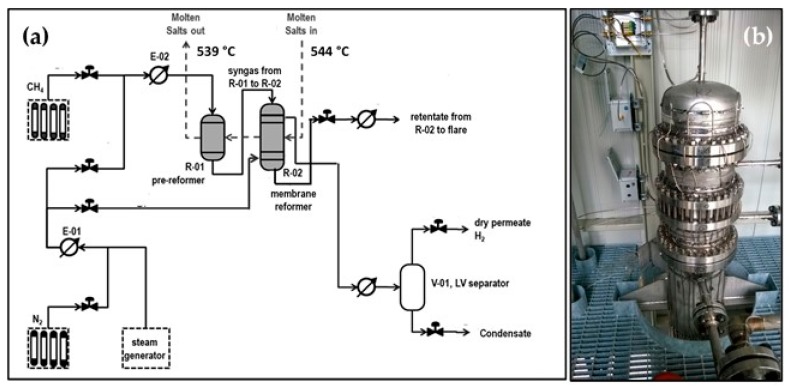
Pure hydrogen production membrane reactor in closed architecture (**a**) Process scheme; (**b**) Reactor assembly.

The closed architecture was tested at pilot level and at a capacity of 3 Nm^3^/h of pure hydrogen (facility available at ENEA Casaccia, Italy, EU Comethy project). The process scheme of the pilot unit is reported in Figure 2 [25,26,27].

The plant architecture is based on a first pre-reformer stage (R-01) followed by an integrated membrane reactor (R-02). The main characteristics of the reactors are reported in Table 4. Methane is supplied by cylinders, while process steam and sweep steam are generated by a dedicated electrical boiler. The reaction heat was supplied through a molten salt mixture fed to R-01 at a maximum inlet temperature of 550 °C and further routed to the R-01. Thanks to the partial conversion carried out in R-01, syngas fed to R-02 contained a certain amount of hydrogen, allowing the membrane to be active just at the entrance of the reactor. R-01 is organized as a shell-and-tube configuration, where the molten salts mixture flows in the shell side supplying reaction heat, and the catalyst is installed inside the tubes. R-02 is also arranged in a shell-and-tube configuration, with the molten salts flowing on the shell side. Catalyst and membrane are arranged according to a tube-in-tube configuration, with the catalyst in the annular section around the membranes tube. The latter is equipped with an inner tube to allow the sweep gas to flow in the permeate side. The permeate stream collected from R-02 was cooled down in order to easily separate the sweep gas as a condensate. 

The nickel noble metal-based catalysts deposited on silicon carbide foam were shaped in the form of a cylinder and an annular cylinder for R-01 and R-02, respectively. The catalysts were prepared at the ProCeed Lab of the University of Salerno. A total of 10 Pd-based membranes on ceramic supports were arranged in R-02, developing an overall area of about 0.35 m^2^, whose main characteristics are reported in Table 5. Each membrane had an outside diameter of 14 mm and a length of 80 cm.

To improve the separation efficiency, superheated steam was employed as sweep gas in a countercurrent configuration. R-01, R-02, and piping in contact with the molten salts were electrically traced in order to assure a temperature above the salts’ freezing point during the start-up and shut-down procedures. 

The main operating conditions of the catalytic tests carried out with the integrated membrane reactor are reported in Table 6.

### 2.2. Synthetic Fuel Production

**Figure 3:**
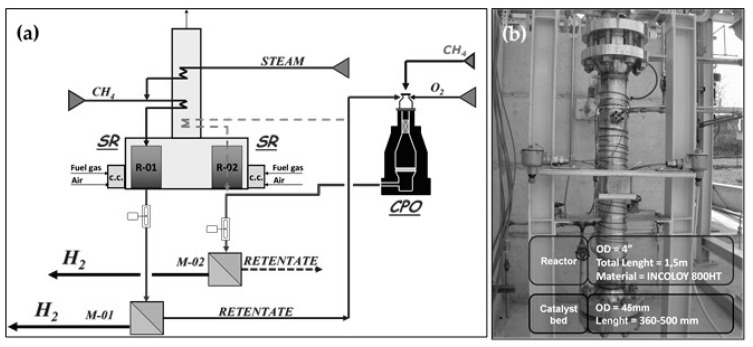
Syngas/pure hydrogen production membrane reactor for gas-to-liquid (GTL) process (**a**) Process scheme; (**b**) Catalytic partial oxidation (CPO) reactor assembly.

The overall concept was tested in open architecture at pilot level and at a capacity of 20 Nm^3^/h of pure hydrogen (facility available at Chieti Scalo, Italy, EU NEXT-GTL project). The process scheme of the pilot unit is reported in Figure 3 [28,29,30].

After desulphurization, the natural gas was mixed with the process steam and preheated in the convective section of the reformer. The preheated steam was further routed to the reforming reactor operated at an outlet temperature of 550–600 °C. The syngas produced was cooled at a temperature in the range of 450–480 °C before entering the first Pd-based membrane module M-01, where a permeate and a retentate stream were produced. The retentate, poor in hydrogen, was routed to the CPO reactor properly mixed inside the reactor with a stream of pure oxygen from the gas cylinders. The hot syngas available at the outlet of the CPO reactor was cooled down to 450–480 °C in a gas–gas heat exchanger and routed to the second Pd-based membrane separator M-02 for a further hydrogen recovery step. The resulted retentate was a syngas, whose composition could be adjusted on the basis of the membrane hydrogen recovery factor. As reported in Figure 3, the CPO reactor could be operated in a standalone mode (with an external CH_4_ stream) or fully integrated with the membrane, accordingly fed with retentate, as described above. Structured catalysts in the forms of honeycomb monolith and pellets, both based on noble metals, were used in the CPO reactor. The main characteristics of the CPO reactor are reported in Table 7.

The membrane-based GTL process was operated at the operating conditions reported in Table 8.

### 2.3. Propylene Production

**Figure 4:**
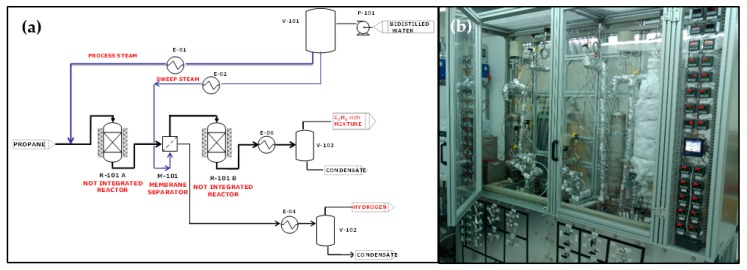
Propylene production membrane reactor (**a**) Process scheme; (**b**) Pilot unit assembly.

The overall concept was tested in open architecture at pilot level and at a capacity of 0.05 kg/h of propylene (facility available at the University of Salerno, Italy, EU CARENA project). The process scheme of the pilot unit is reported in Figure 4 [31,32,33,34].

The experimental apparatus is constituted by two tubular catalytic reactors (R-101 and R-102) and, between them, a selective separation unit (membrane, M-101): such arrangement defines the “open architecture” of the membrane-based process. The catalytic units consist of a catalytic reactor, characterized by a tubular shape and made of AISI 310 stainless steel (SS); the reactor was loaded with a platinum–tin-based catalyst. The catalytic bed loading was also optimized with inters in order to minimize the undesired side reactions occurring in the homogeneous phase, both before and after the catalytic bed (on reactants and products streams). The separation unit is constituted by a Pd-based membrane prepared on an SS porous tube and having an overall permeation surface of 0.01 m^2^. The temperature of the three process devices was controlled by means of electrical heaters, driven by a controller–programmer. The units were then wrapped in a thick layer of insulating mat to minimize heat losses. The catalytic activity tests were carried out in the following operating conditions: Weight Hourly Space Velocity (WHSV) (kg_C3H8_/h·kg_catalyst_) of 8 h^−1^, steam/propane ratio of 0.25 mol/mol, T_reaction_ = 500 °C, P = 5 barg, T_membrane_ = 370 °C.

## 3. Results and Discussion

### 3.1. Pure Hydrogen Production

The behavior of the two-stages reaction and separation based configuration was deeply investigated. The experimental results in terms of membrane stability and feed conversion confirmed, from a technical point of view, the feasibility of the proposed architecture. The development of proper start-up and shut-down procedures, especially with respect to the heating-up and cooling-down sequences, assured a stable operation for the three membrane modules. 

The results clearly evidenced the effect of membrane separation on shifting the reaction towards the product, once hydrogen was removed from the syngas stream, and a second reaction stage was foreseen. 

**Figure 5:**
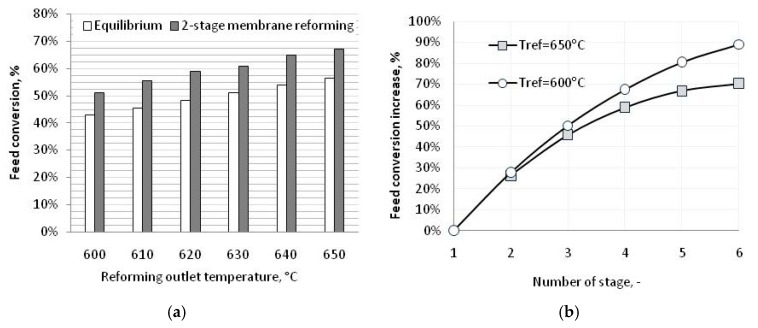
(**a**) Feed conversion for different reforming outlet temperatures (P = 10 barg, Steam/Carbon ratio S/C = 4.3 mol; Tmem = 410–420 °C, Amem = 0.4 m^2^, Molar sweep gas/feed ratio = 0); (**b**) Multistage membrane steam reforming performance (P = 10 barg, S/C 4.3, Tmem = 410–420 °C, Amem = 0.4 m^2^, Molar Sweep gas/feed ratio = 0).

Figure 5a reports the experimental data collected on a two-stage membrane reforming when the M-01A membrane was in operation. With a hydrogen recovery factor in the order of 30–35%, the two-stage configuration allowed to overcome the equilibrium conversion by 20–25%. Hydrogen purity of at least 99.9% mol was detected for all membranes. Because of the modular concept characterizing the open architecture, the performance for a higher number of reaction and separation stages could be easily extrapolated, as shown in Figure 5b. With a number of reaction stages up to six, for example, feed conversion increased up to 70% and 90% at a reformer outlet temperature of 650 °C and 600 °C, respectively. Starting from the experimental results, the behavior of the system for a higher membrane area per stage as well for different values of sweep gas flow rate and reaction pressure could be extrapolated.

An experimental campaign on closed-architecture membrane steam reforming was carried out in order to check the overall system performance. The reforming outlet temperature was regulated by adjusting the inlet molten salts temperature. The latter was limited to 550 °C because of the thermal degradation phenomena occurring in the molten salt mixture at a higher temperature. 

Despite the low reaction temperature, a very high feed conversion was observed due to the effectiveness of hydrogen removal from the reaction environment. Hydrogen purity of at least 99.8%mol was detected during the experimental test. 

**Figure 6:**
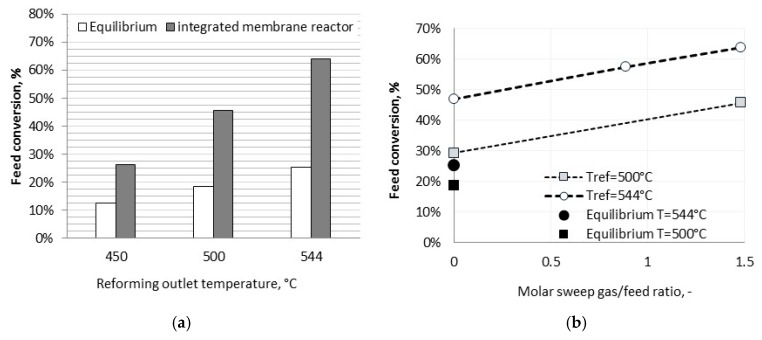
(**a)** Feed conversion at different reforming outlet temperatures (P = 9 barg, S/C = 3 mol and (**b**) Effect of the sweep gas on the integrated membrane reforming plant (P = 9 barg, S/C = 3 mol).

As reported in Figure 6a, the integrated closed configuration allowed to overcome the thermodynamic equilibrium conversion up to 150% under sweep gas condition. The latter plays a major role in the integrated configuration, where H_2_ partial pressure on the reaction side is quite low because of continuous withdrawals. The experimental results showed that, under a molar sweep gas/feed ratio equal to 1.5, the feed conversion increased up to 135–150% with respect to an operating condition without sweep gas. It derived that, by properly adjusting the steam to carbon (S/C) ratio and sweep gas flow rate, a very high feed conversion could be obtained. In a scenario based on the integration of a Concentrated Solar Power (CSP) plant and steam reforming, the operating temperature window of the solar salts mixture (550–290 °C) accounts for a large steam production, thus allowing to optimize the integrated membrane reforming by increasing both the steam/carbon ratio and the sweep gas/feed ratio, without reducing the overall plant efficiency as for a conventional steam reforming [35]. 

The low reaction temperatures combined with a high feed conversion achievable with the integrated configuration allows to minimize the CO content in the retentate side, thus avoiding any post-shift reaction and accounts for a final retentate stream reach in CO_2_ and under pressure. With respect to a conventional steam reformer where the overall produced CO_2_ is available diluted and at atmospheric pressure in the flue gas stream, the proposed architecture allows for a less energy-intensive CO_2_ capture, due to the fact that it is available at s higher partial pressure.

Although more complex from a technologic point view, requiring a new reactor design with respect to conventional one, being able to house a catalyst, a membrane, and sweep gas, the integrated membrane configuration benefits from a higher effectiveness in equilibrium shifting. The contextual hydrogen removal and production allow for a higher hydrogen recovery factor characterizing the integrated membrane architecture, which definitively means a higher feed conversion.

### 3.2. Synthetic Fuel Production

**Figure 7:**
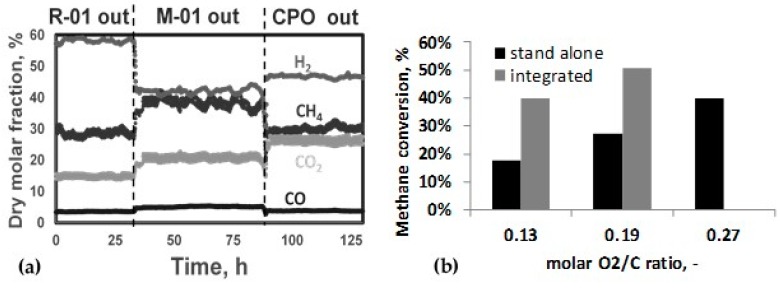
Performance of the membrane-based GTL process: (**a**) Stability, (**b**) Oxygen consumption.

The most relevant results in this application are reported in Figure 7a,b.

Figure 7a shows the product composition on a dry basis at steady-state conditions measured at the outlet of reformer, the membrane separation, and the CPO reactor, respectively. The data were collected when operating the reformer at an outlet temperature of 590 °C, P = 11 atm, with a hydrogen Recovery Factor (HRF) of 45% and with a ratio O_2_/(CO + CH_4_) at the inlet of the CPO reactor of 0.16. The oxygen to carbon ratio was evaluated by taking into account the carbon contribution of CO and CH_4_ in the retentate stream. 

A significant reduction in hydrogen content was observed in the retentate stream due to hydrogen recovery carried out by the first-stage membrane, whereas the concentration of other components increased, since the mixture became more concentrated. In addition, the performance of the membrane system resulted stable for more than 100 h of continuous operation.

Looking at the economics of the novel process for syngas production, it can be observed that the solution consisting of the CPO reactor integrated with the reformer and the membrane enables for a reduction in oxygen consumption, since a portion of feed conversion is achieved in the upstream reformer stage. The removal of hydrogen in the first membrane module has the double role to favor both reactions of partial oxidation of methane and steam reforming inside the CPO reactor. The second membrane module installed downstream of the CPO reactor enables for an additional recovery of hydrogen and might be also used to adjust the final H_2_/CO ratio. In Figure 7b, the comparison of oxygen consumption is reported, observed with both standalone and integrated configuration. The oxygen consumption was referred to the natural gas available at the inlet of the reaction scheme in order to have a direct comparison.

It is possible to observe that, if a total feed conversion of 40% is taken into account, the integrated architecture allows to reduce the oxygen consumption over 50%. This can be translated into a lower operating temperature for the CPO section, accordingly with a difference in the outlet gas composition. The achieved reduction of oxygen consumption enables for a reduction of about 10% in the variable operating cost.

### 3.3. Propylene Production

**Figure 8:**
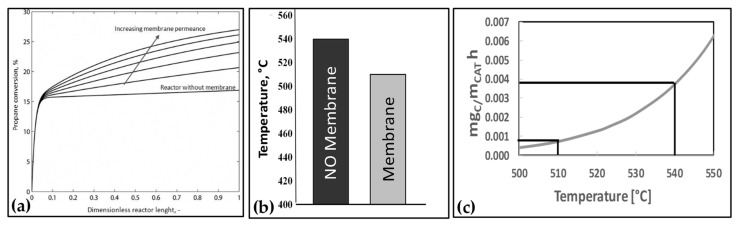
Performance of membrane-based propane dehydrogenation: (**a**) numerical simulation of propane conversion at T = 550 °C and different membrane permeances 40–80 Nm^3^/hm^2^bar^0.5^, (**b**) experimental operating temperature at fixed propane conversion (X_C3H8_ = 10%, 5 barg, S/C3 = 0.25), (**c**) coke amount as a function of the operating temperature.

To proper investigate the membrane-based propylene production process, a dedicated computational model was developed in Matlab environment, aiming to combine the kinetics expression relevant to the main reaction as derived from the literature [36], heat and material balance, and hydrogen permeance law. In Figure 8a, the results of the numerical simulation of propane dehydrogenation reaction coupled with membrane separation are reported, whereas, in Figure 8bc, the experimental results at pilot level are reported. With reference to Figure 8a, the results are reported by indicating, for different values of the membrane permeance, the evolution of the conversion of propane along the catalytic bed. It can be observed that, with a membrane permeance of 40 Nm^3^/(m^2^hbar^0.5^) at 550 °C, it was possible to increase the propane conversion of 35%. The improvement of such performance became 48% when the membrane permeance was increased up to 80 Nm^3^/(m^2^hbar^0.5^).

The results reported in Figure 8b show the temperature at which it was possible to reach a propane conversion of 10% without and with the membrane. In particular, in the absence of the membrane, it was necessary to reach 540 °C to achieve such conversion level. This temperature decreased of about 30 °C when a membrane reactor was used. This achievement could be translated into a lower deposition of carbon matter on the catalytic bed. Indeed, under the assumption of a deactivating model for the catalyst, developed on the basis of the experimental results achieved, it was possible to evaluate that the coke deposited was reduced five times when the membrane was applied (Figure 8c). 

The overall results reported up to now, show the effective applicability of Pd-based membrane reactors in a very large number of chemical processes, even if the actual industrial implementation is strongly related to the possibility of having a stable membrane performance for a long period (at least about 10,000 h) and a cheap cost. 

Indeed, while in the case of hydrogen production (both pure and for GTL applications) the application is in a more advance phase, even if it is still necessary to demonstrate the membrane stability on the long term, for propane dehydrogenation, potential applications might require a further developmental step. In this latter case, in fact, it is much more desirable to limit the membrane operation at temperatures not higher than 400 °C, after a decline in hydrogen flux is observed, owing to the tendency of propylene to form oligomers and ring structures on the membrane that can reduce the performance [37]. This would mean that an industrial application of this concept should take into account such requirement. 

## 4. Conclusions

The development of a chemical industry characterized by resource efficiency, in particular with reference to energy use, is becoming a major issue and driver for the achievement of a sustainable chemical production. From an industrial point of view, several application areas, where energy saving and CO_2_ emissions still represent a major concern, can take benefit of the application of membrane reactors.

Different markets for membrane reactors applications have been analyzed in this paper, and the technical feasibility verified by proper experimentation at pilot level for: (i) pure hydrogen production; (ii) synthetic fuels production; (iii) chemicals production. The achieved results showed that membrane reactors can be effectively used in all mentioned applications.

In most of the proposed solutions, the concept of membrane reactor is based on the application of a sequence of reaction–separation–reaction units rather than on the application of a reactor in which catalyst and membrane are installed in a same vessel according to the concept of process intensification. Indeed, the former solution is still an efficient solution for the overcoming of chemical equilibrium but is characterized by a lower compactness degree compared to the latter. However, in particular applications where the optimal operating conditions for catalyst and membrane operation are so different from each other and the engineering solution to cope with them may become more complex, thereby impacting also on the operation and maintenance of the reaction system, the former solution might represent a faster approach to boost industrial acceptance in the first phase of transition to the novel catalytic membrane reactors technology.

Nevertheless, it is worth to mention that, in order to progress with the industrial implementation of membrane reactors, a few issues need to be solved, such as the reduction of the fabrication costs, the improvement of membrane stability under poisoning, the current lack of industry-produced, commercial-scale units, and the identification of acceptable accelerated ageing tests, without which the membrane stability could be only assessed by carrying out tests for thousands of hours.

## 5. Patents

Iaquaniello, G., Salladini, A. Method for hydrogen production, EP Patent Application 11150491.6, 10 January 2011.

Iaquaniello, G., Salladini, A., Morico, B. Method and system for the production of hydrogen. US Patent US9493350B2, 15 November 2016 (priority date 16 March 2012).

Palo, E., Iaquaniello, G. Method for olefins production. US Patent 9,776,935B2, 3 October 2017 (Priority date 29 March 2011)

## Figures and Tables

**Table 1:** Main characteristics of steam reforming reactors and catalyst.

Element	R-01/R-02
Type	Single tube
Tube Nominal Diameter, in	2 ^1/2^
Tube Active length, m	3
Reactor Volume, L	9
Catalyst shape	Cylinder
Catalyst ID-L, mm-mm	58–150
Catalyst Support material, -	SiC
Catalyst Support porosity, %	87
Catalyst Support pore density, ppi	15

**Table 2:** Main characteristics of the employed membranes.

Membrane	Supplier	Support	Membr. Selective Layer	Thick. Selective Layer, μm	Memb. Area, m^2^	Geometry	Production Method	Permeance, Nm^3^/(hm^2^bar^0.5^) @ 400 °C
M-01/A	ECN	Al_2_O_3_	Pd	3–6	0.4	Tubular	Electroless deposition	32–35
M-01/B	MRT	SS	Pd/Ag	25	0.6	Planar	Proprietary	8–11
M-02	Japanese Company	Al_2_O_3_	Pd/Ag	2–3	0.13	Tubular	Electroless deposition	22–24

**Table 3:** Main operating conditions for two-stage reaction and separation configuration.

Description	I Separation Stage	II Separation Stage
**Syngas Flowrate**		
Overall flowrate, kg/h	25–50	25–50
**Syngas Composition**		
CH_4_, mol%	7–15%	4–8%
CO, mol%	1–3%	1.5–3.5%
CO_2_, mol%	6–7%	7–8.5%
H_2_, mol%	30–36%	27–36%
H_2_O, mol%	42–56%	48–56%
**Pressure (P)**		
P feed side, barg	10.2–10.8	10–9.8
P permeate side, barg	0.4–0.6	0.4–0.6
**Temperature**		
Membrane temperature, °C	350–440	380–440

**Table 4:** Main characteristics of closed-architecture membrane reactors.

Element	R-01	R-02
Type	Shell & Tube	Shell & Tube
Nominal Tube Diameter, in	1	1 ^1/2^
Active length, mm	400	800
N. tube	7	10
Total Reactor Volume, L	1.6	10
Catalyst shape	Cylinder	2-half hollow cylinder
Support material, -	SiC	SiC
Catalyst, -	Pt-Rh	Pt-Ni

**Table 5:** Main characteristics of membranes tested under an integrated reactor.

Membrane	Supplier	Support	Membr. Selective Layer	Thick. Selective Layer, m	Membr. Area, m^2^	Geometry	Production Method	Permeance, Nm^3^/(hm^2^bar^0.5^) @ 400–450 °C
M (R-02)	ECN	Al_2_O_3_	Pd	3–6	0.35	Tubular	Electroless Deposition	10–15

**Table 6:** Main operating conditions for the integrated membrane reactor.

Description	Value
**Flowrate**	
CH_4_ inlet pre-reforming reactor R-01, kg/h	0.3–1.5
H_2_O inlet pre-reforming reactor R-01, kg/h	4.5–6.0
H_2_O Sweep gas, kg/h	0–2.0
Molten salts, kg/h	1250–1650
**Pressure**	
P feed side, barg	9.8–9.5
P permeate side, barg	0.4
**Temperature**	
T range molten salts, °C	450–550

**Table 7:** Main characteristics of the CPO reactor.

Element	Description
Type	Reactor
Nominal Diameter, in	4
Active length, mm	500
Total Active Reactor Volume, l	0.9
Catalyst shape	Cylinder/pellets
Support material, -	Confidential
Catalyst, -	Confidential

**Table 8:** Main operating conditions of the membrane-based GTL process.

Description	Value
**Gas Flowrate**	
CH_4_ inlet reforming reactor R-01, kg/h	6–10
H_2_O inlet reforming reactor R-01, kg/h	18–30
O_2_ inlet CPO reactor, kg/h	1.5–7
**Pressure**	
P, barg	10.5–10.2
T inlet CPO reactor, °C	280–300
T outlet CPO reactor, °C	650–750
